# Is COVID-19 Similar in Pregnant and Non-Pregnant Women?

**DOI:** 10.7759/cureus.8888

**Published:** 2020-06-28

**Authors:** Mohamed Selim, Sherif Mohamed, Manal Abdo, Azza Abdelhaffez

**Affiliations:** 1 Obstetrics and Gynaecology, Zagazig University, Zagazig, EGY; 2 Chest Diseases and Tuberculosis, Faculty of Medicine, Assiut University, Assiut, EGY; 3 Obstetrics and Gynaecology, Mataria Teaching Hospital, Cairo, EGY; 4 Medical Physiology, Assiut University, Assiut, EGY

**Keywords:** covid-19, better outcomes, clinical features, human pathophysiology, cell-mediated immunity, adaptive immunity, complications’, ards, covid-19 in pregnancy

## Abstract

Approximately one-third of infected pregnant women died from severe acute respiratory syndrome coronavirus (SARS-CoV) and the Middle East respiratory syndrome coronavirus (MERS-CoV) epidemics of the past two decades. It is logical to predict that pregnant women infected with the novel coronavirus (SARS-CoV-2) might be at higher risk for severe illness, morbidity, or mortality compared with non-pregnant women. However, a review of the literature indicates that pregnant women are not more likely to be seriously ill than other healthy non-pregnant women if they develop coronavirus disease (COVID-19). This observation begs the question: “Why does pregnancy not increase the risk for acquiring SARS-CoV-2 infection, nor does it worsen the clinical course of COVID-19 compared with non-pregnant individuals?” Herein, we try to explain our observations when considering whether the immunologic changes of pregnancy and other physiologic adaptations of pregnancy affect the virulence and course of SARS-CoV-2 infection.

## Introduction and background

It is logical to predict that pregnant women infected with severe acute respiratory syndrome coronavirus 2 (SARS-CoV-2) causing coronavirus disease (COVID-19) might be at higher risk for severe illness, morbidity, or mortality compared with non-pregnant women. However, available data suggest that pregnancy and childbirth do not increase the risk for acquiring SARS-CoV-2 infection and do not worsen the clinical course of COVID-19 compared with non-pregnant individuals of the same age, and most infected mothers recover without undergoing delivery [1,2]. On the other hand, some pregnant patients with severe COVID-19 have laboratory evidence of an exuberant inflammatory response (similar to cytokine release syndrome), which has been associated with critical and fatal illnesses [3]. These observations create a dilemma: “Why does pregnancy not increase the risk of acquiring SARS-CoV-2 infection, nor does it worsen the clinical course of COVID-19 compared with non-pregnant individuals?” Herein, we explain our observations when considering whether the immunologic changes of pregnancy and other physiologic adaptations of pregnancy affect the virulence and course of SARS-CoV-2 infection.

## Review

Clinical virology

The main clinical features of COVID-19 are mild upper respiratory tract infection with fever, cough, typical ground-glass opacities in radiographic studies, lower respiratory tract infection involving non-life-threatening pneumonia, and life-threatening pneumonia with acute respiratory distress syndrome (ARDS) [4]. SARS-CoV-2, the virus causing COVID-19, is a novel betacoronavirus (large RNA virus) that shares 80% sequence homology with the earlier SARS-CoV virus that caused the SARS outbreak in 2003 [5]. However, SARS-CoV-2 has evolved several features that make it a more efficient than SARS-CoV. The most critical receptor-binding domain of SARS-CoV-2 preserved the overall configuration of the SARS-CoV binding domain, including eight of the 14 residues being completely identical [6]. However, the 3D structure of the SARS-CoV-2 binding site shows that it is more compact, has improved binding stability, and potentially enhanced angiotensin-converting enzyme 2 (ACE-2) receptor-binding affinity [6]. Another important difference is that SARS-CoV-2 contains a polybasic (furin) cleavage site inserted at the boundary of the S1/S2 subunits of the spike S-protein [7,8]. This furin-binding site is a feature shared by several recent highly pathogenic viruses, including avian influenza, and can enhance the virus’ ability to internalize into cells.

Pregnancy immune response

Pregnancy is considered as a unique immunological state in which the maternal immune system has to face two opposing challenges: establishing and maintaining tolerance to the contained allogeneic fetus while keeping the capability to protect against different microbial threats. Finely tuned immune adaptations should be perfect, in order to maintain this neutral immune status. It was observed that the maternal immunological states actively adapt and dynamically change with the development of the growing fetus: from a proinflammatory state in early pregnancy to an anti-inflammatory state in mid-pregnancy, and finally reaching a rather proinflammatory state in late pregnancy [9]. During pregnancy, pathophysiologic adaptations play a role, so the upper respiratory tract tends to be swollen due to high levels of estrogen and progesterone, and restricted lung expansion makes the pregnant woman more susceptible to respiratory pathogens as well [10].

COVID-19 and pregnancy interaction

Previous studies have shown that SARS infection during pregnancy can lead to high rates of spontaneous abortion, premature birth, and intrauterine growth restriction. However, there was no evidence of vertical transmission from the mother to the child [11]. Therefore, these complications may be attributed to the direct effect of the virus on mothers. However, this scenario seems to be different in SARS-CoV-2. Severe symptoms such as pneumonia and marked hypoxia are widely described with COVID-19 in older people, the immunosuppressed, and those with long-term comorbidities, such as diabetes, cancer, and chronic lung disease. These could occur in pregnant women, yet the absolute risks of SARS-COV2 infection during pregnancy are, however, small [1,2,4,5]. A case series from New York of 43 women who tested positive for COVID-19 showed a pattern of disease severity similar to non-pregnant adults [12]. Other reported cases of COVID-19 pneumonia in pregnancy were milder and had good recovery [4,5]. On a darker note, a cytokine storm in COVID-19 has been recently described. In severe cases, COVID-19 infection is associated with a cytokine storm, which causes increased plasma concentrations of interleukin (IL) 2, IL-7, IL-10, granulocyte-colony stimulating factor (G-CSF), interferon-γ-inducible protein 10, monocyte chemoattractant protein 1, macrophage inflammatory protein 1 alpha, and tumor necrosis factor α. This storm is the primary mechanism for ARDS responsible for mortality in patients with severe COVID-19 [3]. Previous reports had shown that patients with SARS had preferential activation of T helper (Th)-1 immunity which led to markedly elevated levels of proinflammatory cytokines (IFNγ, IL-1β, IL-6, and IL-12), which in turn leads to extensive lung damage. In contrast, patients with COVID-19 demonstrated activation of both Th1 and Th2 immunity, culminating inexpression of IFNγ, IL-1β, IL-4, and IL-10 [13]. It was observed that pregnancy increases influenza-related effects through several mechanisms, including disrupted viral clearance, increased pulmonary expression of IL-6, IL-1α, and G-CSF, and enhanced physiological stress in the lungs, influenced by changes in prostaglandin and progesterone levels [14]. However, in COVID-19, a range of immune responses have been described, and early adaptive immune responses may be predictive of milder disease severity [15]. Thus, hormonal changes in pregnancy that influence immunological responses to viral pathogens, together with the physiological transition to a Th2 environment favoring the expression of anti-inflammatory cytokines (IL-4 and IL-10) and other unidentified immune adaptations, may serve as the predominant immune response to SARS-CoV-2, resulting in the lesser severity of COVID-19 [13,15].

A possible role for ACE-2 expression in COVID-19 and pregnancy interactions

The structure of the SARS-CoV-2 receptor-binding gene region is very similar to that of SARS-CoV, and both viruses use the same receptor for cell entry, the ACE-2. While the expression of ACE-2 occurs predominantly within type II alveolar cells of the lung, the receptor is also present in several extrapulmonary sites, including the heart, kidney, and the endothelium [16]. The renin-angiotensin system (RAS) is a crucial regulator of systemic hemodynamics. In healthy human pregnancy, the vasoconstrictor peptide angiotensin II (Ang II) is elevated; however, there is a counterbalancing vasodilator effect by angiotensin-(1-7) [Ang-(1-7)], which is seen increased in both plasma and urine [17]. ACE-2 is the main Ang-(1-7) generating enzyme and affects the relative expression and/or functions of Ang II and Ang-(1-7). It is thought that Ang-(1-7) contributes to the reduction in the systemic vascular resistance and the maintenance of normal systemic blood pressure, as evidenced by its defective expression in preeclampsia [18]. Previous studies had demonstrated the positive correlation of ACE-2 expression and infection of SARS-CoV in vitro [19]. Several ACE-2 variants could reduce the association between ACE-2 and S-protein in SARS-CoV. Therefore, susceptibility and clinical outcomes of SARS-CoV-2 infection might be tightly related to the expression level and pattern of human ACE-2 in different tissues. Asian males might have a higher expression of ACE-2, as denoted by a recent single-cell RNA-sequencing (RNA-seq) analysis [20]. East Asian populations have much higher allele frequency in the expression quantitative trait loci variants associated with higher ACE-2 expression in tissues, which may suggest different susceptibility and/or response to SARS-CoV-2 from diverse populations under similar conditions [21]. Therefore, the severity of COVID-19 in pregnant women could be related to the attenuated expression of ACE-2 in those patients.

## Conclusions

Several pathophysiologic and immunologic mechanisms could impact viewing the scenario of COVID-19 in pregnancy. However, the question of why pregnancy does not worsen the clinical course of COVID-19 remains. Although the answer is not clear, some mechanisms could explain. The unique immunologic changes of pregnancy may suppress virus virulence, pregnancy causes some changes in ACE-2 receptor modulation and expression that may interfere with virus pathophysiology, and maternal adaption in lung tissue prevents severe lung injury by the presence of extra steroid hormones that may interfere with the fat content of the virus envelope. COVID-19 is still new, and a large amount of information is still being explored. Further studies are warranted.
